# Spatial ecology of a wastewater network defines the antibiotic resistance genes in downstream receiving waters

**DOI:** 10.1016/j.watres.2019.06.075

**Published:** 2019-10-01

**Authors:** Marcos Quintela-Baluja, M. Abouelnaga, Jesus Romalde, Jian-Qiang Su, Yongjie Yu, Mariano Gomez-Lopez, Barth Smets, Yong-Guan Zhu, David W. Graham

**Affiliations:** a School of Engineering, Newcastle University, Newcastle upon, Tyne, UK; b Department of Analytical Chemistry, Nutrition and Food Science, School of Veterinary Sciences, University of Santiago de Compostela, Lugo, Spain; c Departamento de Microbiología y Parasitología, Universidade de Santiago de Compostela, Santiago de Compostela, Spain; d Key Lab of Urban Environment and Health, Institute of Urban Environment, Chinese Academy of Science, Xiamen, China; e Labaqua, Santiago de Compostela, Spain; f Department of Environmental Engineering, Technical University of Denmark, 2800, Kgs. Lyngby, Denmark; g State Key Lab of Urban and Regional Ecology, Research Center for Eco-environmental Sciences, Chinese Academy of Sciences, Beijing, China

**Keywords:** Wastewater networks, Wastewater treatment plants, Antibiotic resistance, Spatial ecology, Microbiomes, Resistomes

## Abstract

Wastewater treatment plants (WWTPs) are an effective barrier in the protection of human and environment health around the world, although WWTPs also are suggested to be selectors and-or reservoirs of antibiotic resistance genes (ARGs) before entering the environment. The dogma about WWTPs as “ARG selectors” presumes that biotreatment compartments (e.g., activated sludge; AS) are single densely populated ecosystems with elevated horizontal gene transfer. However, recent work has suggested WWTP biotreatment compartments may be different than previously believed relative to antibiotic resistance (AR) fate, and other process factors, such as bacterial separation and specific waste sources, may be key to ARGs released to the environment. Here we combined 16S rRNA metagenomic sequencing and high-throughput qPCR to characterise microbial communities and ARGs across a wastewater network in Spain that includes both community (i.e., non-clinical urban) and hospital sources. Contrary to expectations, ARGs found in downstream receiving waters were not dominated by AS biosolids (RAS), but more resembled raw wastewater sources. In fact, ARGs and microbial communities in liquid-phase WWTP effluents and RAS were significantly different (Bray–Curtis dissimilarity index = 0.66 ± 0.11), with a consequential fraction of influent ARGs and organisms passing directly through the WWTP with limited association with RAS. Instead, ARGs and organisms in the RAS may be more defined by biosolids separation and biophysical traits, such as flocculation, rather than ARG carriage. This explains why RAS has significantly lower ARG richness (47 ± 4 ARGs) than liquid-phase effluents (104 ± 5 ARGs), and downstream water column (135 ± 4 ARGs) and river sediments (120 ± 5 ARGs) (Tukey's test, p < 0.001). These data suggest RAS and liquid-phase WWTP effluents may reflect two parallel ecosystems with potentially limited ARG exchange. As such, ARG mitigation in WWTPs should more focus on removing bacterial hosts from the liquid phase, AR source reduction, and possibly disinfection to reduce ARG releases to the environment.

## Highlights

• Microbiomes and ARGs were quantified across a wastewater network in Spain.
• Hospitals contributed highest levels of ARGs/bacterial cell to the network.
• Treatment plant effluent microbiomes resembled influents, not activated sludge.
• Biosolids separation processes primarily controlled ARGs found in receiving waters.
• Operating strategies for ARG mitigation in wastewater networks must be reconsidered.

## Graphical abstract

**Figure:**
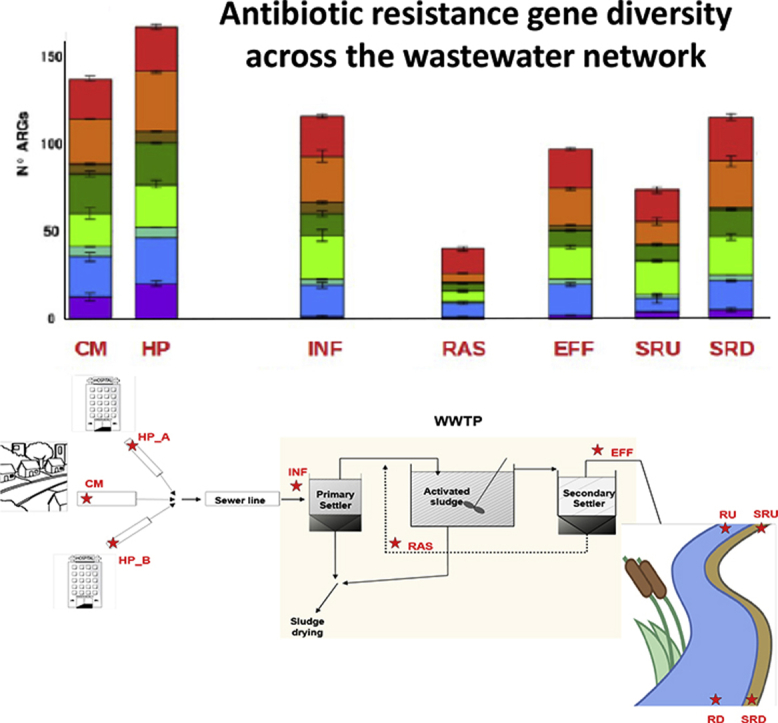

## 1 Introduction

Antibiotics historically have been among the most effective classes of therapeutic drugs used in the treatment of infectious bacterial disease. However, successful treatment has been compromised by increasing antibiotic tolerance or resistance (AR) in bacteria. The ability of microbes to resist some antibiotics is natural, but AR evolution and spread has accelerated in recent years due to widespread use of antibiotics in medicine, agriculture, and aquaculture (Knapp et al., 2010). In terms of spread, domestic wastewater releases are a key link between human gut and environmental microorganisms, influencing the distribution and abundance of antibiotic resistance genes **(ARG)** across aquatic compartments and microbial communities. This has implications to human health owing to possible horizontal gene transfer **(HGT)** between environmental bacteria and human pathogens, impacting the potential evolution and selection of new AR phenotypes.

Wastewater treatment plants **(WWTPs)** are considered as possible selectors and reservoirs of ARGs since WWTPs have abundant microbial communities and receive human-associated microorganisms from hospital and community (non-clinical urban) sources (Bouki et al., 2013; Yang et al., 2013; Guo et al., 2017; Karkman et al*.* 2018). However, dogma about ARG fate in WWTPs has presumed that biotreatment compartments (e.g., activated sludge; **AS**) are single ecosystems with elevated HGT, which recent work suggests may not be correct. In fact, wastewater networks are comprised of a series of different ecosystems (including each WWTP unit operation), although few studies have considered multiple ecosystems when assessing the fate of ARGs in wastewater networks (Li et al*.* 2015). The “spatial ecology” of wastewater networks is more diverse than many realise, comprised of at least four distinct different evolutionary ecosystems that might impact ARG fate and spread. Examples include the gut and faeces of the original individual; the sewer line that carries wastewater to the WWTP; each unit operation within the WWTP; and different receiving water compartments (e.g., water column and sediments). Each of these ecosystems has different antibiotic/chemical exposures, microbial cell densities and diversity, levels of mixing, and meta-habitat conditions; all of which potentially influence resident ARGs, their hosts, and HGT within the overall network.

Here we characterised microbiomes and resistomes across an urban wastewater network in Spain. This network includes community wastes (non-clinical sources), wastes from two hospitals, wastewater treatment in an AS WWTP, and final discharge into a river. Studying a network with two hospitals is important because antibiotic use is more intensive in hospital settings, especially last resort antibiotics, selecting for AR bacteria **(ARB)** over susceptible counterparts (Stalder et al., 2014; Rodriguez-Mozaz et al*.* 2015; Escudero-Onate et al., 2017; Rowe et al*.* 2017; Szekeres et al*.* 2017). Previous studies show hospital-associated wastewaters can contain high levels of resistance to specific antibiotics (Jakobsen et al., 2008; Yang et al*.* 2009; Fuentefria et al*.* 2011; Korzeniewska et al*.* 2013; Hocquet et al*.* 2016), although relative masses and volumes often are low compared with community sources (Li et al., 2015; Hocquet et al., 2016). Despite this, evidence exists that hospital and community resistomes differ and might contribute differently to downstream environmental resistomes (Jakobsen et al*.* 2008; Rita et al., 2013; Picão et al., 2013; Korzeniewska and Harnisz, 2013, Korzeniewska et al., 2013; Rodriguez-Mozaz et al*.* 2015).

As such, we looked holistically at the spatial distribution, bacterial associations, and diversity of ARGs across an entire wastewater network by comparing microbiomes and resistomes among compartments. The goal was to clarify which ecosystems and in-process mechanisms most strongly impact ARGs found in downstream receiving waters to develop better-informed WWTP mitigation solutions for reducing AR releases to the natural environment.

## 2 Material and methods

### 2.1 Study site and sampling

**Fig. 1:**
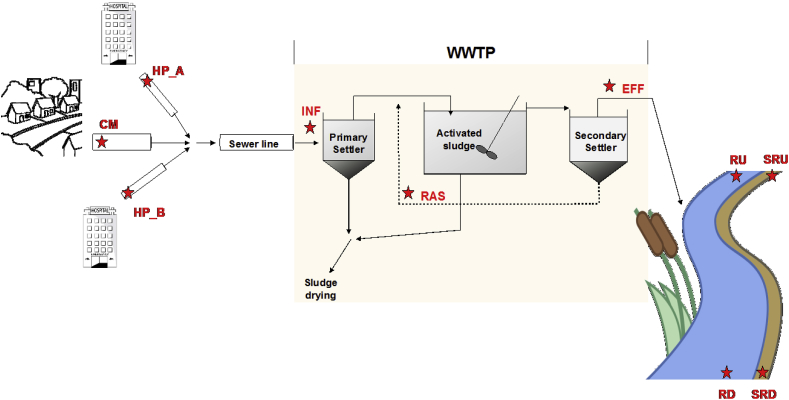
Study area and sampling sites. Label definitions as follows: CM = community wastewater, HP = hospital wastewater (HP_A and HP_B), INF = WWTP influent, RAS = recycled activated sludge, EFF = WWTP liquid effluent, RD = downstream river water column, RU = upstream river water column, SRD = sediment river downstream, and SRU = sediment river upstream.

Sampling was performed in summer 2015 across the wastewater network with minimal industrial and agricultural contributions for a city in northwest Spain with an estimated population of 95.800 inhabitants. Summer sampling was selected to assess the worst-case scenario in terms of dilution of WWTP effluents in receiving waters. The sampling network is shown as Fig. 1. Samples were collected from the sewage effluent from two main hospitals (HP_A and HP_B), community sewage only (CM), and from the influent (INF), liquid effluent (EFF) and recycled activated sludge (RAS) of the municipal WWTP as well as water column and sediments 100 m upstream (RU and SRU) and downstream (RD and SRD) of the WWTP discharge point.

This WWTP was designed to treat 184,000 population equivalents, which equates to an average daily flow of 54,560 m^3^. The receiving river has a width/depth (W/D) ratio of 4.31 and a channel slope of 0.008 m/m. The flow rate during the sampling was estimated at 0.2–0.3 m^3^/s, which was about half the WWTP flowrate during the sampling period (0.4–0.5 m^3^/s). Such dilution is common in southern Europe in the summer (Keller et al., 2014); therefore, this network provides data relevant to any location with limited wastewater dilution, which recent work has found to be important ^7^. The city's two main hospitals (with approximately 1300 beds) contribute less than 2% of volumetric flow to the WWTP. No wastewater treatment is performed at the hospitals. The community sewage was collected before a pumping station in a neighbourhood sewer system of 18,830 habitants.

Sampling was performed when no rainfall had occurred within three days. All sampling included triplicate grab samples per day (n = 3 per location) collected between 9:00 and 11:00 a.m. on week-days over three consecutive work-weeks (2 L), except for river sediment samples, which were collected at the end of the sample campaign at varied locations (n = 6) approximately 100 m downstream and upstream of the WWTP discharge point. From river samples, surface water (5 L) from the top 0.25 m of the water surface, while sediment (∼500 g) was collected from the top 5 cm layer using a gravity-corer.

Samples were collected in sterile polystyrene bottles, transported to the laboratory on ice in coolers within 5 h, and temporarily stored at 4 °C before further analysis. Samples were measured in situ using hand-held probes (Mettler Toledo™, FG3 FiveGo™, and Jenway Model 350 pH Meter) to characterise wastewater conditions, temperature, pH, dissolved oxygen, and conductivity, (Tables S–1).

### 2.2 DNA extraction

DNA was extracted from vacuum-filtered biosolids collected using sterile 0.22-μm membrane disc filters (Millipore, Billerica, MA, USA) or by pelleting via centrifugation at 12,000 rpm for 30 min. Extraction was performed using the Fast DNA Spin Kit for Soils (MP Biomedicals, USA) according to the manufacturer's instructions. DNA was stored at −20 °C prior to subsequent analysis. It was not possible to perform the HT-qPCR for ARGs in samples from the river upstream the WWTP due to low DNA concentration.

### 2.3 16S rRNA gene sequencing and processing

To assess microbial community composition and diversity, PCR amplification of the V4–V5 region of bacterial 16S rRNA genes in DNA extracts was conducted using fusion primers. The primers contained a PGM sequencing adaptor, a “GT” spacer, and a unique 12 base pair Golay barcode to allow multiplex analyses (primers 515F: 5′- GTGNCAGCMGCCGCGGTAA-3′, and 926R: 5′-CCGYCAATTYMTTTRAGTTT-3′). PCR reactions were conducted using the Phusion Flash High-fidelity PCR master mix (ThermoFisher) with the following thermocycle program (i) 10 s denaturation at 98 °C, (ii) 35 cycles of 1 s denaturation at 98 °C, (iii) 5 s annealing at 56 °C, (iv) 15 s elongation at 72 °C, and (v) 1 min elongation at 72 °C. Amplicons were quantified using a Qubit dsDNA HS Assay Kit (Invitrogen) on a Qubit^®^ 2.0 Fluorometer and pooled in equimolar amounts before further purified using a Pippin Prep System (Life Technologies) following the manufacturer's protocol.

Subsequent sequencing was performed using an Ion Torrent Personal Genome Machine (PGM™) System (Life Technology) at Newcastle University. Sequences were processed in UPARSE-QIIME pipeline (Pylro et al., 2014, Pylro et al., 2016). The FastQ files exported from the Ion PGM™ system were analysed following the recommendations of the Brazilian Microbiome Project (BMPOS) (Pylro et al*.* 2016). Briefly, the Operational Taxonomic Unit (OTU) table was built using the UPARSE pipeline (Stalder et al., 2014) in which reads were truncated at 100 bp and quality filtered using a maximum expected error of 0.5. Filtered reads were de-replicated and singletons removed. The sequences were clustered into OTUs at 97% similarity cut-off, checked for chimeras, and representative sequences were obtained for each microbial phylotype (Stalder et al*.* 2014). Taxonomic classification used QIIME (Caporaso et al., 2010) based on the UCLUST method against the Greengenes 13.8 database (Szekeres et al., 2017) with a confidence threshold of 80%.

### 2.4 16S rRNA data analysis and visualization

All data analysis and visualizations used R through the Rstudio IDE (http://www.rstudio.com/) (R Core Team, 2006). OTU counts and associated taxonomic assignments were imported and merged into phyloseq objects (Mcmurdie and Holmes, 2013). All samples were rarefied to ensure the same number of reads per sample (i.e., 8704), which corresponds to the sample with the fewest number of sequences, resulting in 6434 OTUs.

α-diversity indexes (Richness, Simpson and Shannon), pairwise ANOVA of diversity measures between sampling sites, Non-metric multidimensional Scaling (NMDS) ordination, and local contributions to β-diversity all were calculated using the R package microbiomeSeq (Ssekagiri et al., 2017). Good coverage was calculated using the goods function of QsrUtils package. Ranked abundance distribution curves and cluster dendrograms of community composition dissimilarity (Bray-Curtis, average neighbour clustering) were calculated with the R package vegan (Leclercq and Wang, 2016). R package DESeq2 was used to identify significant differences in taxonomic normalized genes at the order level (Love et al., 2014).

### 2.5 Biomarker signature analysis (LefSe)

To determine bacterial taxa with significantly different abundance among sampling sites, biomarker analysis was performed using the linear discriminant analysis (LDA) effect size (LEfSe) method (Segata et al., 2011) in conjunction with an OTU-normalized relative abundance matrix. The LEfSe method uses the Kruskal-Wallis test to identify significant differences and performs an LDA to evaluate the effect of taxa group size. A threshold score of 2 and a significant α of 0.05 were used to detect differences.

### 2.6 Evidence of different wastewater network microbial communities in receiving river microbial communities

**SourceTracker,** a Bayesian approach for estimating proportions of a community containing mixed sources (Knights et al., 2011), was used to estimate the relative contributions of microbial communities from different “sources” across the wastewater network to downstream “sinks” (Leclercq et al*.* 2016; Gou et al., 2018). To perform this analysis, 16S rRNA sequence data were grouped in cluster dendrograms of community composition dissimilarity (Bray-Curtis, average neighbour clustering) based on OTU distributions for characteristic sources. Sources included raw wastewater (e.g., community and hospital wastes, and WWTP influent) (n = 12), RAS (n = 3), and the river upstream (n = 3). To check homogeneity of this source classification, we used “leave-one-out” source-class prediction for Bayesian models to ensure that all identified sources looked the same.

The sinks included the liquid effluent from the WWTP, the downstream water column, and downstream river sediments. OTUs present in only one sample were removed prior to the analysis. SourceTracker uses Gibb's sampling (Markov chain Monte Carlo algorithm) to estimate the source proportions and allocates unexplained OTUs in the sinks as from an “unknown source”. SourceTracker analysis was carried out at a depth of 8,704, with 100 iterations [default], 10 re-starts [default], and used the auto-tuning functionality.

### 2.7 Integrons, total bacteria and coliform quantification

Class 1, 2, and 3 integron gene cassettes were quantified using quantitative PCR (qPCR) (Tables S–2). Taqman qPCR reactions were conducted using SsoAdvanced™ Universal Probes Supermix (BioRad), employing the following thermocycle program: (i) 3 min of initial denaturation at 95 °C, and 40 cycles of (ii) 5 s denaturation at 95 °C, and (iii) 30 s annealing/extension at 60 °C. In addition, qPCR also was used to quantify total eubacteria and coliforms using a SYBR green-based method assay (see Tables S–2). SYBR-green reactions were conducted using SsoAdvanced™ Universal SYBR^®^ Green Supermix (BioRad), employing the following thermocycle program: (i) 2 min of initial denaturation at 98 °C, and 40 cycles of (ii) 5 s denaturation at 98 °C, and (iii) 5 s annealing/extension at 60 °C (total bacteria) or 55 °C (total coliforms).

All assays were done in triplicate using the BioRad CFX C1000 System (BioRad, Hercules, CA USA). In order to avoid inhibitor effects, DNA samples were diluted to a working solution of 5 ng/ul and an internal control DNA always was used in SYBR-green reactions. Standard curves for each set of primers were constructed using plasmid clones of the target sequences of between 10^3^ and 10^8^ copy numbers, which were used in triplicate and in parallel with each qPCR run.

### 2.8 ARGs via high-throughput quantitative PCR (HT-qPCR)

To evaluate the abundance of ARGs in samples, high-throughput qPCR (HT-qPCR) of ARGs was performed using the SmartChip Real-time PCR (Wafergen Inc. USA) as described previously (Wang et al., 2014). A total of 296 primer sets (Tables S–3) were used, including 294 validated primer sets targeting 285 ARGs conferring resistance to major classes of antibiotics, eight transposases and one 16S rRNA gene. HT-qPCR data were pre-processed for each primer set and amplification efficiencies outside an acceptable range (90%–110%) were discarded. Amplification was confirmed with at least two positive replicates.

### 2.9 HT-qPCR and qPCR statistical analysis

Data were processed using the R environment (version 3.4.3, http://www.r-project.org/), while relative copy number of ARG, transposase genes, and integrase genes were calculated and transformed to absolute copy numbers by normalizing to 16S rRNA gene copy numbers for each sample. Based on the Ribosomal RNA Database (Rrndb), the average number of 16S rRNA-encoding genes per bacteria genome (hereinafter referred as “genome”) is estimated as 4.1 (Klappenbach et al., 2001). 16S rRNA-encoding gene quantities were divided by this value to estimate the number of genomes, and the normalized copy numbers of ARG or transposases per genome were calculated.

Statistical analyses and data manipulation were performed using the R environment with a significant cutoff of α = 0.05. Normality was studied by the Shapiro-Wilk test; whereas, homoscedaticity of the variance was assessed using the Levene's test. When previous conditions were met, one-way analysis of variance **(ANOVA)** was performed to assess statistically significant differences and, if applicable, subsequent **Tukey post-hoc test** for pairwise comparisons were performed between sampling site pairs. When datasets failed to meet normality requirements, non-parametric statistical analysis were applied for all comparisons. Thus, a **Krustall-Wallis** test was performed to assess statistically significant differences and, if applicable, subsequent a **Games-Howell post-hoc test** for pairwise comparison between sampling sites were performed.

### 2.10 Correlation analysis between ARG subtypes and bacterial communities

A Mantel test and Procrustes analysis were performed to analyse the relationships between ARGs and bacterial communities. The Mantel test was based on Bray-Curtis dissimilarity matrices of the normalized ARGs and OTUs data, using vegan packages in R. The threshold for significance was p < 0.05. To perform the Procrustes analysis, normalized ARGs and OTUs data were used for non-metric multidimensional scaling (NMDS) analysis (Oksanen, 2015). The two resulting NMDs were compared using the Procrustes function and significance tested using 999 permutations.

### 2.11 Co-occurrence between ARG subtypes and microbial taxa

A correlation matrix was developed by calculating all possible pairwise Spearman's rank correlations among 139 bacterial orders, 149 ARGs subtypes, 5 transposases, and 3 integrases present in samples from the study (n = 27). A correlation between two items was considered statistically robust if the Spearman's correlation coefficient (ρ) was ≥0.8 and the p value was ≤0.01 (Junker and Schreiber, 2008). To reduce the chances of obtaining false-positive results, p values were adjusted with a multiple testing correction using the Benjamini–Hochberg method (1995). The robust pairwise correlations of ARG subtypes formed co-occurrence networks. Network analyses were performed in R, and was visualized and explored to identify its topological properties (i.e., clustering coefficient, shortest average path length, and modularity) in Gephi (Bastian et al., 2009).

## 3 Results

### 3.1 Microbial communities across the wastewater network

Bacterial abundances, expressed as a proportion of 16S rRNA gene copy number per ng of metagenomic DNA, varied by one order of magnitude among samples (1.14× 10^7^ to 1.34 × 10^8^ copies per ng DNA) (Tables S–4), suggesting bacterial cells were a relatively constant proportion of the total biomass. β-diversity analysis was used to compare sample diversity among sites. For this analysis, the dataset was re-sampled to obtain the same number of reads per sample, which was the sample with the fewest number of sequences, resulting in 6434 OTUs in the analysis. The trend of rarefaction curves suggests sufficient representation of the microbial communities (Figure S-1). Good's coverage estimate showed high values, all above 93% (Table 1), indicating our selection of 8704 reads provided a reasonable representation of the sampled communities (Tables S–4)

**Fig. 2:**
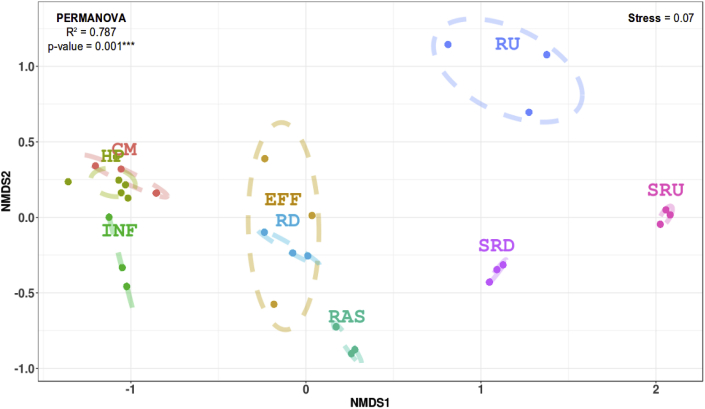
Non-metric multidimensional Scaling (NMDS) of a Bray–Curtis resemblance matrix among 30 samples obtained from wastewater network. Labels are as follows: CM = community wastewater, HP = hospital wastewater (HP_A and HP_B), INF = WWTP influent, RAS = return activated sludge, EFF = WWTP liquid effluent, RD = downstream river water column, RU = upstream river water column, SRD = sediment river downstream, and SRU = sediment river upstream.

Rarefaction curves for OTUs showed different bacterial community diversities across sampling sites, which were confirmed when evaluating α-diversity metrics, including Richness, Shannon and Simpson indices (Figure S-1, Tables S–4). These indices indicate that raw wastewater-associated samples have significantly lower diversity compared with upstream river samples (both water column and sediment), WWTP liquid effluent, and downstream river samples (both water column and sediment) (p-value < 0.05). Therefore, bacterial diversity was greater in non-wastewater samples, presumably due to more rare taxa, which is supported by rank abundance distributions (Figure S-2). Additionally, the Bray-Curtis dissimilarity dendrogram shows the community structure follows a pattern closely defined by wastewater treatment steps (Figure S-3), containing three main clusters (cut-off = 0.72). The first one cluster contains river samples upstream of the WWTP (water column and sediment). The second cluster contains samples associated with raw sewage (community and hospital sewage, and WWTP influent), while the third cluster contains the RAS, WWTP effluent and the downstream river water and sediment samples. Distances among different microbial community structures (β-diversity) were visualized in a NMDS plot (Fig. 2), where one can see that WWTP effluents and microbial communities in the river downstream appear related, although relationships are subtle. For example, WWTP effluent resembles the downstream water column, whereas downstream river sediments more closely align with the RAS.

The composition of bacterial communities also differ among sites at the phylum level (Figure S-4). Proteobacteria and Bacteroidetes are generally prevalent at all sites. Conversely, Firmicutes dominate both community and hospital wastewaters, but are lower in the WWTP influent, RAS, and the upstream river. Chloroflexi and Planctomycetes are particularly evident in river sediments (upstream and downstream), and also in the RAS. Clear differences exist between liquid-phase wastewater (e.g., raw sources and WWTP effluents) and RAS-associated samples. In fact, the RAS microbial composition is very different from other samples, except the downstream river sediments.

### 3.2 Biomarker signature analysis in water sanitation systems

**Fig. 3:**
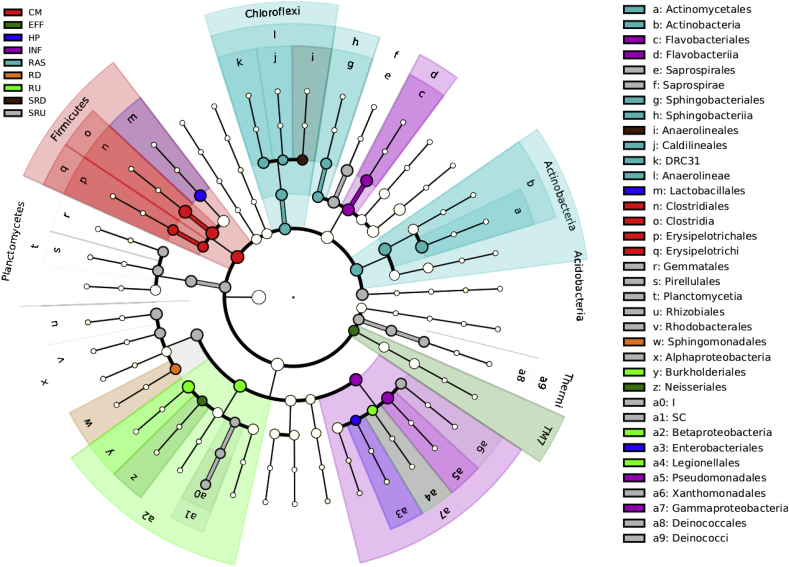
A linear discriminant analysis effect size (LEfSe) method identifies the significantly different abundant taxa of bacteria in all the sampling sites. The taxa with significantly different abundances among sites are represented by coloured dots, and from the center outward, they represent the kingdom, phylum, class, and order. The coloured shadows represent trends of the significantly differed taxa. Each coloured dot has an effect size linear discriminant analysis (LDA) score. Only taxa meeting an LDA significance threshold of >2 are shown. Samples labelled as follows: CM = community wastewater, HP = hospital wastewater (HP_A and HP_B), INF = WWTP influent, RAS = return activated sludge, EFF = WWTP liquid effluent, RD = downstream river water column, RU = upstream river water column, SRD = sediment river downstream, and SRU = sediment river upstream.

Characterising microbial communities in each compartment of a wastewater network (in terms of diversity, evenness, and taxonomic composition) is key to identifying linkages among compartments and microbial contributions from outside sources. We used LEfSE to identify taxa that were differentially present with each compartment versus taxa that might be present in one compartment, but potentially transferred from other compartments. LEfSE analysis showed community wastewater was best characterised by the orders Clostridiales and Erysipelotrichales (Fig. 3). In contrast, hospital wastewater was better characterised by the presence of Lactobacilliales and Enterobacteriales, while Pseudomonadales and Flavobacteriales tend to reflect WWTP influent. RAS was defined by Spingobacteriales, Caldilineales, and Actinomycetales (Fig. 3). As such, each compartment has a selected “characteristic” orders to help delineate the relative influence of different source communities on downstream sink communities.

### 3.3 Effect of wastewater network microbial communities on microbial communities in the receiving river

**Fig. 4:**
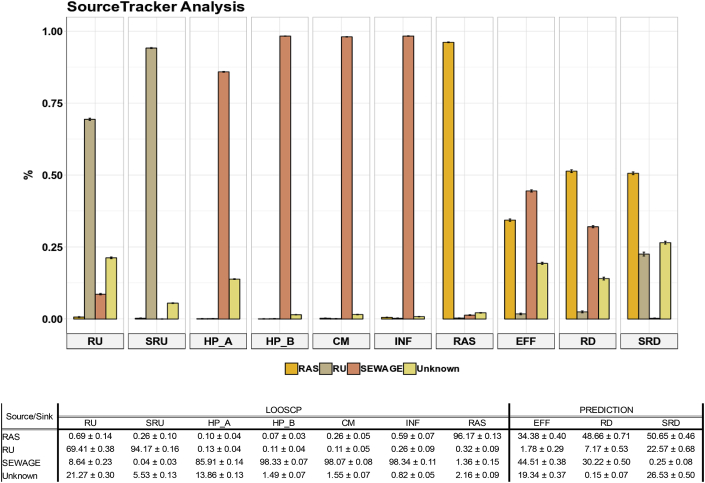
Relative contribution of river upstream sediment and water column, sewage (hospital and community sewage, and influent), RAS, river upstream (water column and sediment), and unknown sources to the wastewater treatment plant effluent and river downstream (water columns and sediment) estimated using SourceTracker analysis. Where CM = community wastewater, HP = hospital wastewater (HP_A and HP_B), INF = WWTP influent, RAS = return activated sludge, EFF = WWTP liquid effluent, RD = downstream river water column, RU = upstream river water column, SRD = sediment river downstream, and SRU = sediment river upstream.

SourceTracker analysis was performed to explore the fate of each source, including raw wastewater (i.e., hospital, community, and WWTP influent), RAS, and upstream river water (Fig. 4, Tables S–5). Each source was quite distinct based on their Bray-Curtis dissimilarity index (Figure S-3), showing the leave-one-out source class prediction provided a reasonable reflection of sources (Fig. 4, Tables S–5). This allows us to proportionate source influences in sinks. For example, sequences in the liquid WWTP effluent microbial community were mainly a mixture of raw wastewater (42% ± 0.41) and RAS bacteria (33% ± 0.34). Similarly, the downstream water column was a combination of raw wastewater bacteria (30% ± 0.5) and RAS (49% ± 0.71). In contrast, downstream sediment sequences were different, being dominated by RAS (51% ± 0.54) and upstream river sediment bacteria (16% ± 0.59), showing less influence of raw sewage (<0.4%). Downstream sediments were dominated by RAS bacteria, whereas the water column was more influenced by WWTP influent bacteria. Finally, upstream communities (water column and sediment) were largely substituted downstream by bacteria from the WWTP (Fig. 4, Tables S–5).

The Venn diagram (Figure S-5) confirms that raw WWTP influent and RAS have very different OTU compositions and that WWTP effluent is a combination of both, including bacterial species from the WWTP influent that do not appear to establish themselves in RAS. The Figure S-6 summarises relative abundances of effluent taxa associated with the influent (Figure S–6B**)**, RAS (Figure S–6C), and persistent taxa from both (Figure S–6A). Statistically significant differential abundances in microbial taxa (order level) exist between WWTP influent and RAS (Figure S-7), which are especially evident when one compares the WWTP effluents with influents (Figure S–7B) and RAS (Figure S–7C), respectively. In summary, RAS contributes significantly to the presence of Acidimicrobiales, Actinomycetales, Caulobacteriales, Cytophagales, Myxococcales, PHOS-HD20, Rhizobiales, Saprospirales, Sphingobacteriales, and Spingomonadales. Conversely, WWTP influent more contributes to the presence of Aeromonadales, Bacterioidales, Campilobacteriales, Clostridiales, Desulfovibrionales, Enterobacteriales, and Neisseriales.

On a network level, significantly higher relative abundances of Enterobacteriales were found in hospital wastewaters, WWTP influent, and liquid-phase WWTP effluents (compared with the RAS); a conclusion supported by qPCR data on targeted coliform bacteria (Figure S-8). Both raw hospital and community wastewaters have significantly higher relative coliform levels than the RAS, implying coliforms less readily colonise the RAS (see Figure S–6B). This has been suggested before, which is explained by the fact that such organisms tend not to intrinsically flocculate (Huang et al., 2018). In contrast, coliform levels in liquid WWTP effluent are proportionally higher than in RAS. This is further evidence that a sub-community of Enterobacteriales passes directly through the WWTP into the downstream water column.

### 3.4 Richness and relative abundance of ARGs and MGEs in wastewater networks and receiving rivers

**Fig. 5:**
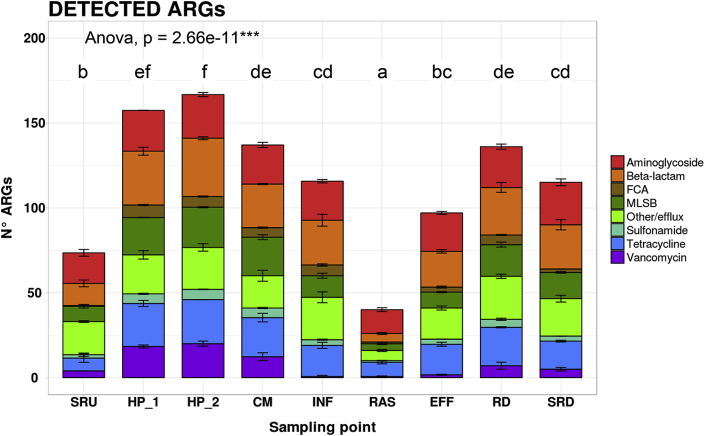
Number of antibiotic resistance genes (ARGs) detected in the sampling sites. Resistance genes are classified based on the antibiotics to which they confer resistance. They include aminoglycosides, β-lactams, FCA (fluoroquinolone, quinolone, florfenicol, chloramphenicol and amphenicol resistance genes), MLSB (macrolide-lincosamide-streptogramin B), other/efflux (multidrug-efflux pumps or others), sulphonamides; tetracyclines; and vancomycin. The statistical analyses, comparing the number of resistance genes in each site were performed using one-way analysis of variance (ANOVA) and post-hoc Tukey test.

A total of 255 ARGs and eight transposase genes were quantified by HT-qPCR, and three integrase genes were quantified by qPCR across all sites. Detected ARGs encode resistance to eight classes of antibiotics, with aminoglycosides, β-lactam, multidrug-efflux pumps, tetracycline, and MLSB resistance being the most frequently encountered types (Fig. 5). Some observations are possible. First, liquid-phase WWTP effluents significantly contributed to the number of detected ARGs in the river, with 122 ARGs found in downstream sediments (significantly greater than the 80 ARGs found in upstream sediments; p-value < 0.01). Additionally, the highest number of ARGs were found in the hospital wastewaters (both HP_A and HP_B, mean = 169 ± 8); this was higher than community wastewater (n = 146 ± 11) and significantly higher than ARGs in the WWTP influent (n = 124 ± 21) (p-value < 0.01) (Tables S–6). The lowest number of ARGs were found in the RAS, which contains only 47 ± 4 ARGs; much less than 104 ± 5 ARGs in the WWTP effluent (see Fig. 5).

Absolute ARG concentrations detected in all samples were high, ranging from 6.16 × 10^8^ (WWTP effluent) to 8.63 × 10^10^ (RAS) copies per ml or gram (Figure S-9). The same was seen for transposon genes with concentrations ranging from 1.01 × 10^7^ (WWTP effluent) to 1.16 × 10^9^ (RAS) copies per gram or ml; and integrase genes ranging from 7.37 × 10^6^ (WWTP effluent) to 2.17 × 10^9^ (RAS) copies per gram or ml. After RAS, the downstream river sediments had the highest concentration of ARGs (5.40 × 10^10^ copies per gram), transposon genes (8.75 × 10^8^ copies per gram), and integrase genes (1.10 × 10^9^ copies per gram). These were significantly higher (p-value < 0.01) than found in the upstream sediments (6.35 × 10^9^ copies of ARGs per g, 7.12 × 10^7^ copies of transposases per g, and 5.96 × 10^8^ copies of integrases per g). The highest ARG abundances were found in hospital wastewaters (HP_A as 3.12 × 10^10^ and HP_B as 2.23 × 10^10^ copies of ARG per ml). These levels are greater than associated 16S rRNA concentrations, suggesting that “hospital bacteria” may carry multiple ARGs per genome (more than from community wastewaters).

ARGs conferring resistance to aminoglycosides were dominant in all samples, increasing in relative abundance from the WWTP influent (0.383 ± 0.042 ARGs/genome) to RAS (0.536 ± 0.365 ARGs/genome). The same pattern is true for genes conferring resistance to FCA, sulphonamides, and vancomycin, although only one gene was detected in the latter two cases (Figure S-10). ARGs conferring resistance to β-lactam antibiotics were the second most abundant type per genome in raw wastewater sources (hospital and community), ranging from 2.649 ± 0.349 ARGs/genome in hospital wastes (HP_A and HP_B) to 0.199 ± 0.044 ARGs/genome in community wastes. By contrast, multidrug-efflux pumps were the second most common mechanism, ranging from 0.273 ± 0.122 ARGs/genome in WWTP effluents to only 0.037 ± 0.001 ARGs/genome in upstream sediments. Less abundant gene classes were for FCA, ranging from 0.177 ± 0.027 ARGs/genome in hospital wastes (HP_A and HP_B) to 0.009 ± 0.008 ARGs/genome in the WWTP effluent. Finally, vancomycin resistance genes were 0.037 ± 0.006 ARGs/genome in hospital wastes (HP_A and HP_B), but were less than 0.001 ARGs/genome in WWTP influent.

Although the WWTP itself significantly reduces the concentrations of most ARG groups (between influent and effluent; Tables S–7), actual ARG richness and the number of resistance genes per genome did not change significantly between the WWTP influent and effluent. Relative to river sediment resistomes, a significant increase in ARG concentrations were seen in all groups upstream and downstream of the WWTP, except for FCA and vancomycin. The richness of ARGs conferring resistance to aminoglycosides, β-lactams, MLSB, and tetracycline all increased significantly (p < 0.01) (Tables S–8). Further, the average number of ARGs per genome in downstream sediments also increased significantly for aminoglycosides, β-lactams, MLSB, multidrug efflux systems, tetracyclines, and also the number of transposase and integrase genes per genome.

Overall, ARGs, transposase, and integrase genes per genome (Tables S–9) were highest in the hospital wastewaters. For example, 13.9 ARGs per genome were detected in hospital wastewaters, which is much higher than community wastewater (1.6 ARGs/genome), RAS (1.0 ARGs/genome), WWTP liquid effluent (0.8 ARGs/genome), upstream river sediments (0.1 ARGs/genome), downstream river sediments (1.4 ARGs/genome), and the downstream water column (0.6 ARGs/genome). In this network, hospital wastewater was only 1.65–1.84% of the total flow volume to the WWTP; however, based on mass balances (assuming 9.39 log of genomes per ml in hospital wastes and 9.28 log of genomes per ml in community wastes), hospital wastes contribute from 15.8 to 17.3% of ARGs to the WWTP. Finally, a Venn diagram overlaying ARGs present in hospital versus community sources and the receiving waters show 15 unique ARGs are attributable to hospitals, whereas only six ARGs are attributable to community wastes (Figure S-11).

Using two-dimensional hierarchical clustering in conjunction with an ARG heatmap of relative abundances (Figure S-12), ARG co-occurrence patterns were delineated across network compartments. Sample types split into general clusters, with hospital wastewater samples clustering together in terms of ARGs, whereas community wastewater more clusters with WWTP influent and effluent, and the downstream water column. In contrast, ARGs in upstream river sediments and the RAS cluster very different from all other samples. Clustering suggests ARGs found in the RAS minimally relate to WWTP influents and downstream water column samples.

### 3.5 Relationships between bacterial communities and ARGs

The Mantel test showed that bacterial community compositions were significantly correlated with ARGs compositions according to the Bray-Curtis dissimilarity index (R = 0.338, P = 0.003). Procrustes analysis further supports significant correlations between prevalent ARGs and bacterial composition (16S rRNA gene OTUs data) (Bray−Curtis dissimilarity index; sum of squares M12 = 0.344, r = 0.810, P = 0.001, 999 permutations) (Figure S-13). These results confirm resistomes generally link with microbial communities. Here, the WWTP influent, liquid-effluent and downstream water column resistomes were similar, whereas RAS was very different.

### 3.6 Co-occurrence patterns among ARGs subtypes

Co-correlation networks are well suited to detecting general patterns in highly populated taxonomic groups. Co-occurrence patterns between ARGs and microbial taxa (order-level) were investigated using a network analysis approach (Figure S-14). We hypothesized that non-random co-occurrence patterns between ARGs and microbial taxa would suggest possible host information of ARGs if the ARGs and co-existing microbial taxa display strong and significantly positive correlations (Spearman's R^2^ 0.8, P < 0.01). In data here, the co-correlation network consisted of 203 nodes (ARG subtypes) and 1593 edges with an average degree or node connectivity of 15.695. The average network distance between all pairs of nodes (average path length) was 2.771 edges with a network diameter of 8 edges. As shown in Figure S-14, network analysis produces two independent groups. The first group (Figure S–14A) includes all ARGs, transposase genes, and integrase genes, and associates with only 13 taxa. In contrast, the second group contains taxa only and no AMR-related elements (Figure S–14B).

**Fig. 6:**
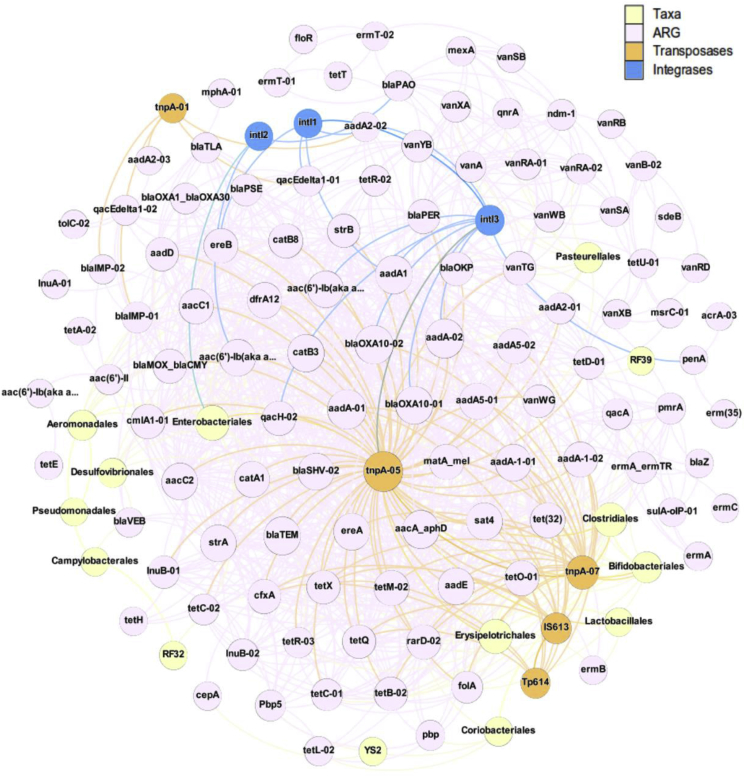
Network analysis revealing co-occurrence patterns among ARG subtypes, taxa (order level), transposons and integrons. A connection represents a strong (Spearman's correlation coefficient p > 0.8) and significant (P-value > 0.01) correlation. The size of each node is proportional to the number of connections, that is, the degree.

Both groups can be visualized as independent networks (see Fig. 6), with the first group as probable ARG hosts with characteristic bacteria from wastewater sources (community, hospital and WWTP influent), including Enterobacteriales, Pseudomonadales, and Clostridiales (Figure S–6A). The second group, which does not correlate with ARGs, transposase genes, or integrase genes (Figure S-15), is primarily composed of RAS-enriched taxa (Figure S–6A), such as Actinomycetales and Spingomonadales. This is further corroboration that the RAS microbial community does not strongly associate with ARGs in WWTP effluents.

## 4 Discussion

This study elucidated the spatial ecology of ARGs within a Southern European wastewater network that includes both hospital and community wastewater sources. The wastewater network was in Spain, sampled during “worst-case scenario” conditions when WWTP effluent dilution rates in the river were very low; a common scenario in drier climates.

Data show this wastewater network can be divided into three microbial source communities (raw wastewater, RAS, and the river upstream), which differentially explain where how and why ARGs spread across the larger network (Baquero et al., 2008). These communities relate to three evolutionary ecosystems with different habitat and selection factors. The first ecosystem and its microbial community are the raw wastewater sources (hospital, community, and WWTP influent). Hospital and community wastewaters are microbiologically closer to raw waste sources (faecal matter) whereas microbial communities change as the wastewater flows down the sewer line. This change is characterised by a shift from obligate anaerobes (presumably from source faeces) to facultative anaerobes (Shanks et al*.* 2013; Bengtsson-Palme et al*.* 2015).

The second ecosystem is the core WWTP biological treatment community (i.e., RAS), which despite continuous inputs of wastewater, has its own unique microbial composition that significantly differs from the wastewater sources and the liquid-phase WWTP effluent. The RAS community has high ARG abundances, but very low ARG richness and does not resemble wastewater sources in terms of ARGs or bacterial composition. Based on this and other data, we suspect ARGs found in RAS are largely coincidental because the RAS community is primarily being selected by WWTP operating conditions, such as biosolids settling within the secondary clarifier. This conclusion is consistent with Cai et al*.* (2014) and Huang et al*.* (2018) who showed microbial communities in activated sludge were less affected by the WWTP influent bacteria, suggesting large fractions of WWTP influent bacteria disappear or decrease significantly in the RAS compared with the influent (Tang et al., 2016). In total, these data suggest that free-living microorganisms, which do not aggregate in flocs or do not readily settle in the clarifier, appear to pass through the WWTP. It further suggests RAS and the liquid-phase (and their microorganisms) may represent two ecosystems, which is key to explaining ARG fate within and beyond the biotreatment compartment. Most studies on AR studies presume WWTPs, especially RAS, are a place of active ARG exchange (e.g., Ma et al., 2011; Burch et al., 2013), which data here suggest may not be the case.

The third ecosystem and its microbial community associated with the upstream river (water column and sediment), which clusters away from the other groups. This community clearly changes upon reception of the WWTP effluents, becoming a combination of microorganisms from the raw wastewater, RAS, and upstream community in the downstream water column and sediments. Although wastewater treatment reduces the bacterial load by several orders of magnitude, large volumes of treated wastewater inputs increase the abundance and richness of ARGs in the river sediment downstream compared to upstream of WWTP discharges. These results are consistent with those of other studies (Pruden et al*.* 2012; Marti et al*.* 2013; Karkman et al., 2016; Brown et al., 2019).

Overall, findings here are consistent with Munck et al. (2015) who showed the core resistome of biological wastewater treatment units is different from other parts of urban water ecosystems and not necessarily a “hot spot” for gene transfer. Specifically, we show human-waste associated ARGs often pass directly through WWTPs without inclusion into the RAS. Therefore, although biological treatment units and RAS are important to carbon and nitrogen removal, other factors are more important to the fate of ARGs within the same WWTPs. Data suggest the type and performance of biosolids separation units may be key to downstream resistomes. The non-floc phase has greater ARG richness and bacteria with more ARGs/genome, suggesting that removing unsettlable biosolids may be more critical for reducing ARG releases to the environment. If this is true, greater emphasis is needed in understanding and improving biosolids separation in WWTPs. Implicitly, membrane bioreactors may be better from removing ARGs, which reports have suggested (Lea et al., 2018; Zhu et al*.* 2018).

## 5 Conclusions

This study shows that understanding the spatial ecology of a wastewater network is critical to explaining what impacts ARGs released from WWTPs. Specifically, RAS and the associated liquid phase in biotreatment compartments appear to be two parallel ecosystems. As such, ARG fate and releases from a WWTP may be more associated with bacterial biophysical traits, such as tendencies towards flocculation and settling. It also shows that source wastewater ARGs may be more important to WWTP effluents than believed, albeit in subtle ways. As an example, greater ARG richness and higher levels of ARGs/genome prevail in hospital sources might disproportionately influence ARGs entering the WWTP and, in turn, organisms passing through the WWTP in liquid effluents to the receiving water. This problem may be particularly acute in southern Europe in the summer or anywhere else where receiving water dilution levels are low.

Taken together, this work shows less studied factors, such as the spatial ecology of whole networks and the local ecology of unit operations, may be critical to improving ARG mitigation by WWTPs. Based the network studied, future focus should be on AR source reduction, improving biosolids separation, and possibly disinfection to reduce ARG releases in the wider environment.

## Declaration of interests

NA.
