# Supplementary material for: Spatial ecology of a wastewater network defines the antibiotic resistance genes in downstream receiving waters
Source: Water Res. 2019 Oct 1;162:347–57. doi: 10.1016/j.watres.2019.06.075 (PMC6650630; doi:10.1016/j.watres.2019.06.075)
Supplement: Multimedia component 1 [file mmc1.doc]

1. Supplementary Information
2. Spatial ecology of a wastewater network defines the antibiotic resistance genes in downstream receiving waters

Marcos Quintela-Baluja^1^, M. Abouelnaga^2^, Jesus Romalde^3^, Jian-Qiang Su^4^, Yongjie Yu^1^, Mariano Lopez Gomez^5^, Barth Smets^6^, Yong-Guan Zhu^4,7^, David W. Graham^1^*

^1^School of Engineering, Newcastle University, Newcastle upon Tyne, UK^#^

^2^Department of Analytical Chemistry, Nutrition and Food Science, School of Veterinary Sciences,University of Santiago de Compostela, Lugo. Spain

^3^Departamento de Microbiología y Parasitología. Universidade de Santiago de Compostela, Santiago de Compostela. Spain

^4^Key Lab of Urban Environment and Health, Institute of Urban Environment, Chinese Academy of Science, Xiamen, China

^5^Labaqua, Santiago de Compostela, Spain

^6^Department of Environmental Engineering, Technical University of Denmark, 2800 Kgs. Lyngby, Denmark

^7^State Key Lab of Urban and Regional Ecology, Research Center for Eco-environmental Sciences, Chinese Academy of Sciences, Beijing, China

*Corresponding author: Prof David W. Graham

School of Engineering

Cassie Building

Newcastle University

Newcastle upon Tyne

United Kingdom NE1 7RU

Phone: +44-191-208-7930

**E-mail:** [**david.graham@newcastle.ac.uk**](mailto:david.graham@newcastle.ac.uk)

**Table S-1:** Catchment metadata from the different sites. Measured values are from three different weeks per site for pH, conductivity, temperature, COD, DNA concentration and bacteria density.

**Table S-2:** qPCR primer and probes list used in this study.

**Table S-3:** HT-qPCR primer list used in this study.

**Table S-4:** *Bacterial abundances, expressed as a proportion of 16S rRNA gene copy number per ng of metagenomic DNA (log of 16S rRNA per ng of DNA). Three α-diversity metrics: observed number of OTUs (richness), and Shannon and Simpson diversity indices as well as the Good’s coverage estimate are included in the table.*

**Table S-5:** SourceTracker analysis. The column “Analysis” reflects results coming from “leave-one-out” source class prediction for Bayesian models to make sure that all sources within each class look the same (LOOSCP) or if they are the prediction of sources in each sink (PREDICTION). The mean and the standard deviation (Sd) are estimations of the OTUs coming from each source in each sink after 100 iterations and 10 re-starts.

| Source tracker results | | | | |
| --- | --- | --- | --- | --- |
| Analysis | Source | Sink | mean | Sd |
| LOOSCP | RAS | CM | 0.01 | 0.00 |
| LOOSCP | RAS | CM | 0.00 | 0.00 |
| LOOSCP | RAS | CM | 0.00 | 0.00 |
| LOOSCP | RAS | HP_A | 0.00 | 0.00 |
| LOOSCP | RAS | HP_A | 0.00 | 0.00 |
| LOOSCP | RAS | HP_A | 0.00 | 0.00 |
| LOOSCP | RAS | HP_B | 0.00 | 0.00 |
| LOOSCP | RAS | HP_B | 0.00 | 0.00 |
| LOOSCP | RAS | HP_B | 0.00 | 0.00 |
| LOOSCP | RAS | INF | 0.01 | 0.00 |
| LOOSCP | RAS | INF | 0.01 | 0.00 |
| LOOSCP | RAS | INF | 0.00 | 0.00 |
| LOOSCP | RAS | RAS | 0.97 | 0.00 |
| LOOSCP | RAS | RAS | 0.96 | 0.00 |
| LOOSCP | RAS | RAS | 0.96 | 0.00 |
| LOOSCP | RAS | RU | 0.01 | 0.00 |
| LOOSCP | RAS | RU | 0.00 | 0.00 |
| LOOSCP | RAS | RU | 0.01 | 0.00 |
| LOOSCP | RAS | SRU | 0.00 | 0.00 |
| LOOSCP | RAS | SRU | 0.00 | 0.00 |
| LOOSCP | RAS | SRU | 0.00 | 0.00 |
| LOOSCP | RU | CM | 0.00 | 0.00 |
| LOOSCP | RU | CM | 0.00 | 0.00 |
| LOOSCP | RU | CM | 0.00 | 0.00 |
| LOOSCP | RU | HP_A | 0.00 | 0.00 |
| LOOSCP | RU | HP_A | 0.00 | 0.00 |
| LOOSCP | RU | HP_A | 0.00 | 0.00 |
| LOOSCP | RU | HP_B | 0.00 | 0.00 |
| LOOSCP | RU | HP_B | 0.00 | 0.00 |
| LOOSCP | RU | HP_B | 0.00 | 0.00 |
| LOOSCP | RU | INF | 0.00 | 0.00 |
| LOOSCP | RU | INF | 0.00 | 0.00 |
| LOOSCP | RU | INF | 0.00 | 0.00 |
| LOOSCP | RU | RAS | 0.00 | 0.00 |
| LOOSCP | RU | RAS | 0.00 | 0.00 |
| LOOSCP | RU | RAS | 0.00 | 0.00 |
| LOOSCP | RU | RU | 0.46 | 0.00 |
| LOOSCP | RU | RU | 0.84 | 0.00 |
| LOOSCP | RU | RU | 0.79 | 0.00 |
| LOOSCP | RU | SRU | 0.95 | 0.00 |
| LOOSCP | RU | SRU | 0.96 | 0.00 |
| LOOSCP | RU | SRU | 0.92 | 0.00 |
| LOOSCP | WASTEWATER | CM | 0.97 | 0.00 |
| LOOSCP | WASTEWATER | CM | 0.98 | 0.00 |
| LOOSCP | WASTEWATER | CM | 0.99 | 0.00 |
| LOOSCP | WASTEWATER | HP_A | 0.99 | 0.00 |
| LOOSCP | WASTEWATER | HP_A | 0.99 | 0.00 |
| LOOSCP | WASTEWATER | HP_A | 0.60 | 0.00 |
| LOOSCP | WASTEWATER | HP_B | 0.99 | 0.00 |
| LOOSCP | WASTEWATER | HP_B | 0.97 | 0.00 |
| LOOSCP | WASTEWATER | HP_B | 0.98 | 0.00 |
| LOOSCP | WASTEWATER | INF | 0.98 | 0.00 |
| LOOSCP | WASTEWATER | INF | 0.98 | 0.00 |
| LOOSCP | WASTEWATER | INF | 0.99 | 0.00 |
| LOOSCP | WASTEWATER | RAS | 0.01 | 0.00 |
| LOOSCP | WASTEWATER | RAS | 0.01 | 0.00 |
| LOOSCP | WASTEWATER | RAS | 0.02 | 0.00 |
| LOOSCP | WASTEWATER | RU | 0.20 | 0.00 |
| LOOSCP | WASTEWATER | RU | 0.01 | 0.00 |
| LOOSCP | WASTEWATER | RU | 0.05 | 0.00 |
| LOOSCP | WASTEWATER | SRU | 0.00 | 0.00 |
| LOOSCP | WASTEWATER | SRU | 0.00 | 0.00 |
| LOOSCP | WASTEWATER | SRU | 0.00 | 0.00 |
| LOOSCP | Unknown | CM | 0.02 | 0.00 |
| LOOSCP | Unknown | CM | 0.02 | 0.00 |
| LOOSCP | Unknown | CM | 0.01 | 0.00 |
| LOOSCP | Unknown | HP_A | 0.01 | 0.00 |
| LOOSCP | Unknown | HP_A | 0.01 | 0.00 |
| LOOSCP | Unknown | HP_A | 0.40 | 0.00 |
| LOOSCP | Unknown | HP_B | 0.01 | 0.00 |
| LOOSCP | Unknown | HP_B | 0.02 | 0.00 |
| LOOSCP | Unknown | HP_B | 0.01 | 0.00 |
| LOOSCP | Unknown | INF | 0.01 | 0.00 |
| LOOSCP | Unknown | INF | 0.01 | 0.00 |
| LOOSCP | Unknown | INF | 0.01 | 0.00 |
| LOOSCP | Unknown | RAS | 0.02 | 0.00 |
| LOOSCP | Unknown | RAS | 0.03 | 0.00 |
| LOOSCP | Unknown | RAS | 0.02 | 0.00 |
| LOOSCP | Unknown | RU | 0.33 | 0.00 |
| LOOSCP | Unknown | RU | 0.15 | 0.00 |
| LOOSCP | Unknown | RU | 0.16 | 0.00 |
| LOOSCP | Unknown | SRU | 0.05 | 0.00 |
| LOOSCP | Unknown | SRU | 0.04 | 0.00 |
| LOOSCP | Unknown | SRU | 0.08 | 0.00 |
| PREDICTION | RAS | EFF | 0.53 | 0.00 |
| PREDICTION | RAS | EFF | 0.08 | 0.00 |
| PREDICTION | RAS | EFF | 0.43 | 0.01 |
| PREDICTION | RAS | RD | 0.55 | 0.00 |
| PREDICTION | RAS | RD | 0.59 | 0.00 |
| PREDICTION | RAS | RD | 0.41 | 0.01 |
| PREDICTION | RAS | SRD | 0.46 | 0.00 |
| PREDICTION | RAS | SRD | 0.59 | 0.00 |
| PREDICTION | RAS | SRD | 0.47 | 0.01 |
| PREDICTION | RU | EFF | 0.01 | 0.00 |
| PREDICTION | RU | EFF | 0.03 | 0.00 |
| PREDICTION | RU | EFF | 0.02 | 0.00 |
| PREDICTION | RU | RD | 0.03 | 0.00 |
| PREDICTION | RU | RD | 0.04 | 0.00 |
| PREDICTION | RU | RD | 0.01 | 0.00 |
| PREDICTION | RU | SRD | 0.26 | 0.01 |
| PREDICTION | RU | SRD | 0.17 | 0.01 |
| PREDICTION | RU | SRD | 0.25 | 0.01 |
| PREDICTION | WASTEWATER | EFF | 0.34 | 0.00 |
| PREDICTION | WASTEWATER | EFF | 0.66 | 0.00 |
| PREDICTION | WASTEWATER | EFF | 0.33 | 0.00 |
| PREDICTION | WASTEWATER | RD | 0.25 | 0.00 |
| PREDICTION | WASTEWATER | RD | 0.33 | 0.01 |
| PREDICTION | WASTEWATER | RD | 0.39 | 0.00 |
| PREDICTION | WASTEWATER | SRD | 0.00 | 0.00 |
| PREDICTION | WASTEWATER | SRD | 0.00 | 0.00 |
| PREDICTION | WASTEWATER | SRD | 0.00 | 0.00 |
| PREDICTION | Unknown | EFF | 0.12 | 0.00 |
| PREDICTION | Unknown | EFF | 0.23 | 0.00 |
| PREDICTION | Unknown | EFF | 0.22 | 0.00 |
| PREDICTION | Unknown | RD | 0.17 | 0.00 |
| PREDICTION | Unknown | RD | 0.05 | 0.00 |
| PREDICTION | Unknown | RD | 0.20 | 0.01 |
| PREDICTION | Unknown | SRD | 0.28 | 0.00 |
| PREDICTION | Unknown | SRD | 0.24 | 0.01 |
| PREDICTION | Unknown | SRD | 0.28 | 0.00 |

**Table S-6:** *Multiple comparisons of Means (Tukey contrast). Pairwise comparison of the number of detected ARG. Samples are grouped by sampling site: CM) Community wastewater; HP) Hospital wastewater (combined HP_A and HP_B); INF) Influent WWTP; EFF) Effluent WWTP; RAS) Recycled activated sludge; RU) Water column river upstream; SRU) Sediment river upstream; RD) Water column river downstream; SRD) Sediment river downstream.*

**Table S-7:** Comparison of the concentration (left), number of detected genes by ARG groups (center), and the genes per bacterial genome (right) in WWTP influent and effluent. Significant differences between sediments were tested in pairwise comparisons using the Tukey post-hoc test (normal distributed data) or *Games-Howell post-hoc test (non-normal distributed data)*. The asterisk in the p-value column indicates the level of significance (0.05*, 0.001**, 0.001***). The trend column indicates if the concentration, diversity or number of genes per bacteria genome increased (↑), decreased (↓), or was not significantly different (-).

**Table S-8:** Comparison of the concentration (left), number of detected genes by ARG groups (center), and genes per bacteria genome (right) in the sediment river upstream (SRU) and downstream (SRD). Significant differences between sediments were tested in pairwise comparisons using the Tukey post-hoc test (normal distributed data) or *Games-Howell post-hoc test (non-normal distributed data)*. The asterisk in the p-value column indicate the level of significance (0.05*, 0.001**, 0.001***). The trend column indicates if the concentration, diversity or number of genes per bacteria cell increased (↑), decreased (↓),or it was not significantly different (-).

**Table S-9:** Normalized number of resistance genes, transposases and integrases per bacterial genome from the different sampling sites. On the basis of the Ribosomal RNA Database (Rrndb), the average number of 16S rRNA-encoding genes per bacterium is currently estimated at 4.1. 16S rRNA-encoding gene quantities were thus divided by this value to estimate bacterial genomes.

**Figure S-1:** *Rarefaction curve for OTUs from different sampling sites. Three* *α-diversity metrics: observed number of OTUs (richness), Shannon, and Simpson diversity indices shown as boxplots.*  *RU) water column river upstream, SRU) sediment river upstream, CM) community-associated wastewater, HP) hospital associated wastewater, INF) STP Influent, RAS) STP recycled activated sludge, EFF) STP effluent, RD) water column river downstream, SRD) Sediment river downstream. Pair-wise ANOVA of diversity measures between sampling sites were performed. The asterisk in the figure indicate the level of significance (0.05*, 0.001**, 0.001***).*

**Figure S-2:** Species rank–abundance distributions (dominance-diversity) from each sample. The plots show logarithmic species abundances against species rank order. All samples show a strongly left-skewed curve, with a few dominant OTUs and a tail of rare OTUs. The rank–abundance curves for upstream river samples (both water column and sediment), and the sediment from downstream river samples show longer, flatter tails, suggesting more rare OTUs in those samples. Samples: RU) water column river upstream, SRU) sediment river upstream, CM) community-associated wastewater, HP) hospital associated wastewater, INF) STP Influent, RAS) STP returning activated sludge, EFF) STP effluent, RD) water column river downstream, SRD) Sediment river downstream.


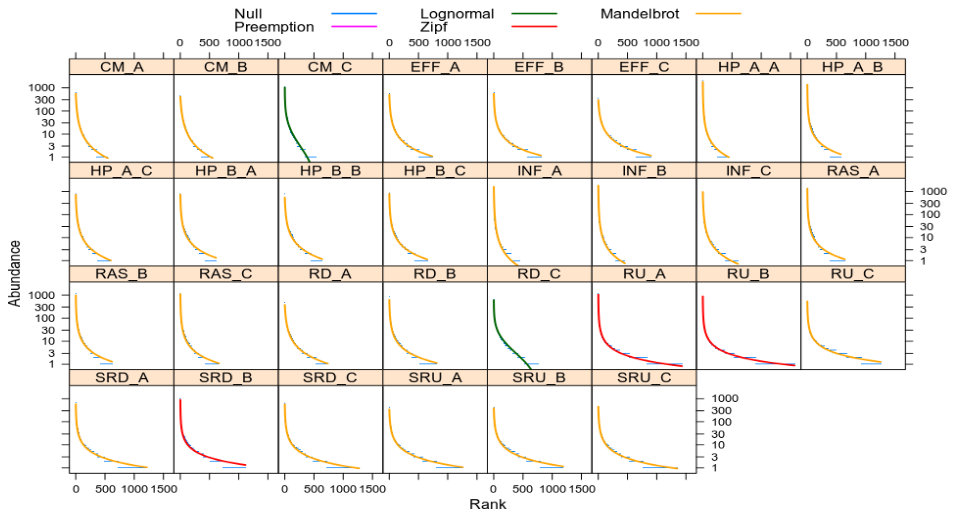


***Figure S-3:*** *Cluster dendrogram of community composition dissimilarity (Bray-Curtis, average neighbour clustering) based on OTU distributions among all sampling sites. The dendogram shows three different clusters at 0.76 cut-off: Cluster A) water column and sediments upstream; Cluster B) community-associated and hospital wastewater, and WWTP Influent; and Cluster C) RAS, WWTP effluent, and water column and sediments river downstream.*

**Figure S-4:** Relative abundance of the dominant bacterial phyla across sample sites. Others; indicate the collective percentage of minor phyla. Sample codes as follows: RU) water column river upstream, SRU) sediment river upstream, CM) community-associated wastewater, HP) hospital associated wastewater, INF) STP Influent, RAS) recycled activated sludge, EFF) STP effluent, RD) water column river downstream, SRD) Sediment river downstream. Degree of uniqueness of a given sample to the variation in community composition was measure by the local contribution to β-diversity (LCBD).

**Figure S-5:** Venn diagram comparing persistent OTU among WWTP influent (INF; n= 208), RAS (n = 330), and WWTP effluent (EFF; n =265). Persistent OTUs are those that were present in all three samples.


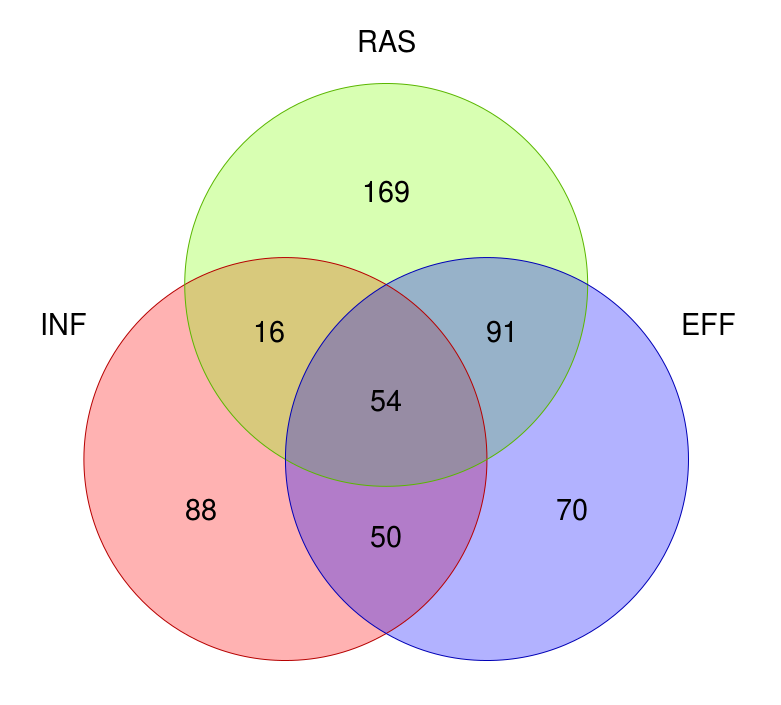


**Figure S-6:** Relative abundance of persistent OTUs at Order level. A) Relative abundance of the 54 OTUs persistent in Influent, RAS and Effluent. B) Relative abundance of50 OTUs persistent in the Influent and Effluent, but not persistent in RAS. C) Relative abundance of 91 OTUs persistent in the RAS and Effluent, but not persistent in Influent.

**Figure S-7.** DESeq2 differential abundance analyses expressed as Log2FC comparison**.** A) Negative Log2FC represent orders enhanced in the Influent (pale red), while positive Log2FC represent orders enhanced in the RAS. B) Negative Log2FC represent orders enhanced in the Influent (pale red), while positive Log2FC represent orders enhanced in the Effluent. C) Negative Log2FC represent orders enhanced in the RAS (pale red), while positive Log2FC represent orders enhanced in the Effluent. Only those orders with p-adj <0.05 are shown.

**Figure S-8:** Relative abundance of coliform bacteria relative to total bacteria number by qPCR assay. Sample codes RU) water column river upstream, SRU) sediment river upstream, CM) community-associated wastewater, HP) hospital associated wastewater, INF) WWTP Influent, RAS) recycled activated sludge, EFF) WWTP effluent, RD) water column river downstream, SRD) Sediment river downstream. The dotted line is the mean of the population, and asterisks indicate if the mean of the samples is enriched or lowered respect to the population mean. Letters indicate distinct groupings based on post-hoc Tukey's-test.

**Figure S-9:** Concentration of 16S rRNA gene, Coliforms, total targeted ARGs, transposases, and integrons in sampling sites (log of copies per ml or g). Samples include: SRU) sediment river upstream, CM) community-associated wastewater, HP) hospital associated wastewater, INF) WWTP Influent, RAS) recycled activated sludge, EFF) WWTP effluent, RD) water column river downstream, SRD) Sediment river downstream.

**
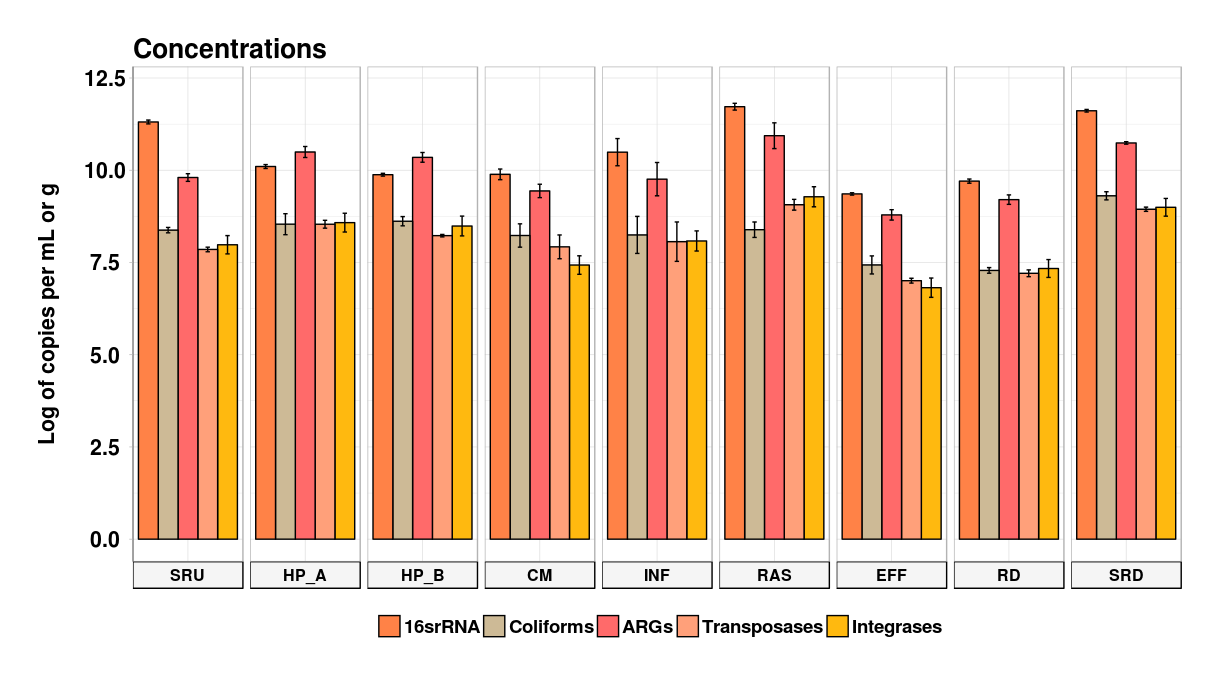
**

**Figure S-10:** Antibiotic resistance genes per bacterial cell (sqrt of ARGs per bacterial cell). Genes conferring resistance to aminoglycosides, β- lactams, MLSB (macrolide-lincosamide-streptogramin B), multidrug-efflux pumps, FCA (fluoroquinolone, quinolone, florfenicol, chloramphenicol and amphenicol resistance genes), sulfonamides, tetracycline, vancomycin, multidrug-efflux pumps or others. Sample codes: SRU) sediment river upstream, CM) community-associated wastewater, HP) hospital associated wastewater, INF) WWTP Influent, RAS) recycled activated sludge, EFF) WWTP effluent, RD) water column river downstream, SRD) Sediment river downstream.

**
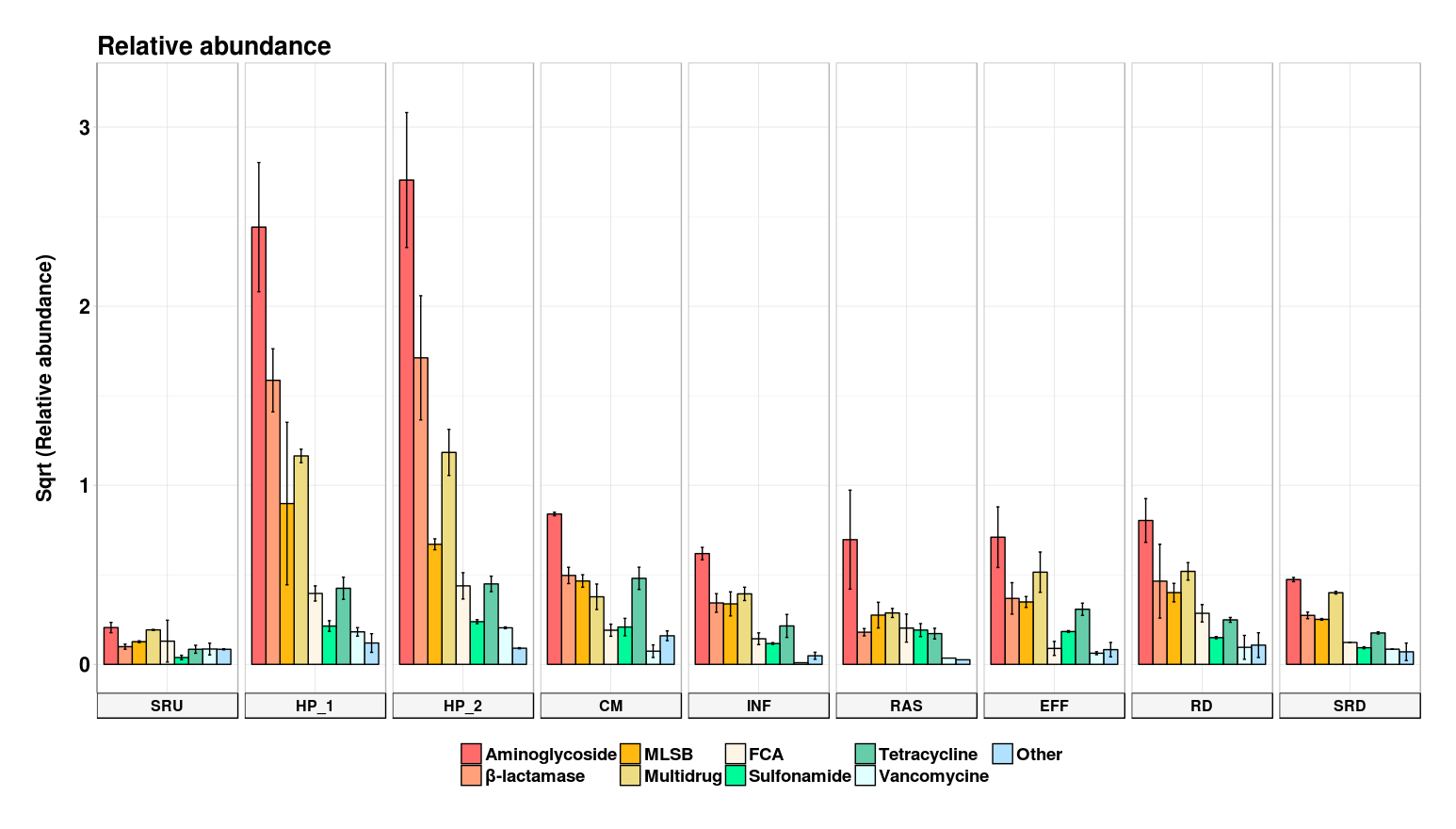
**

**Figure S-11:** *Venn diagram showing the distribution of the ARGs between the wastewater source (hospital versus community), and the receiving river (water column river upstream-RU, sediment river upstream-SRU, and sediment river downstream-SRD).*

**Figure S-12:** Heatmap and cluster analysis (Bray–Curtis; complete linkage) of ARGs in the different sampling points; relative abundances are log-transformed for visualization purposes. ARGs (x-axis) and samples (y-axis) are clustered based on similar abundance and occurrence patterns. Cell colours indicate the relative abundance gradient of the ARGs at each point (red = high abundance, and pale-yellow = no presence). Just genes with a relative abundance of at least 0.1% in one of the samples were used (n=101). Sample codes: SRU) sediment river upstream, CM) community-associated wastewater, HP) hospital associated wastewater, INF) WWTP Influent, RAS) recycled activated sludge, EFF) WWTP effluent, RD) water column river downstream, SRD) Sediment river downstream.


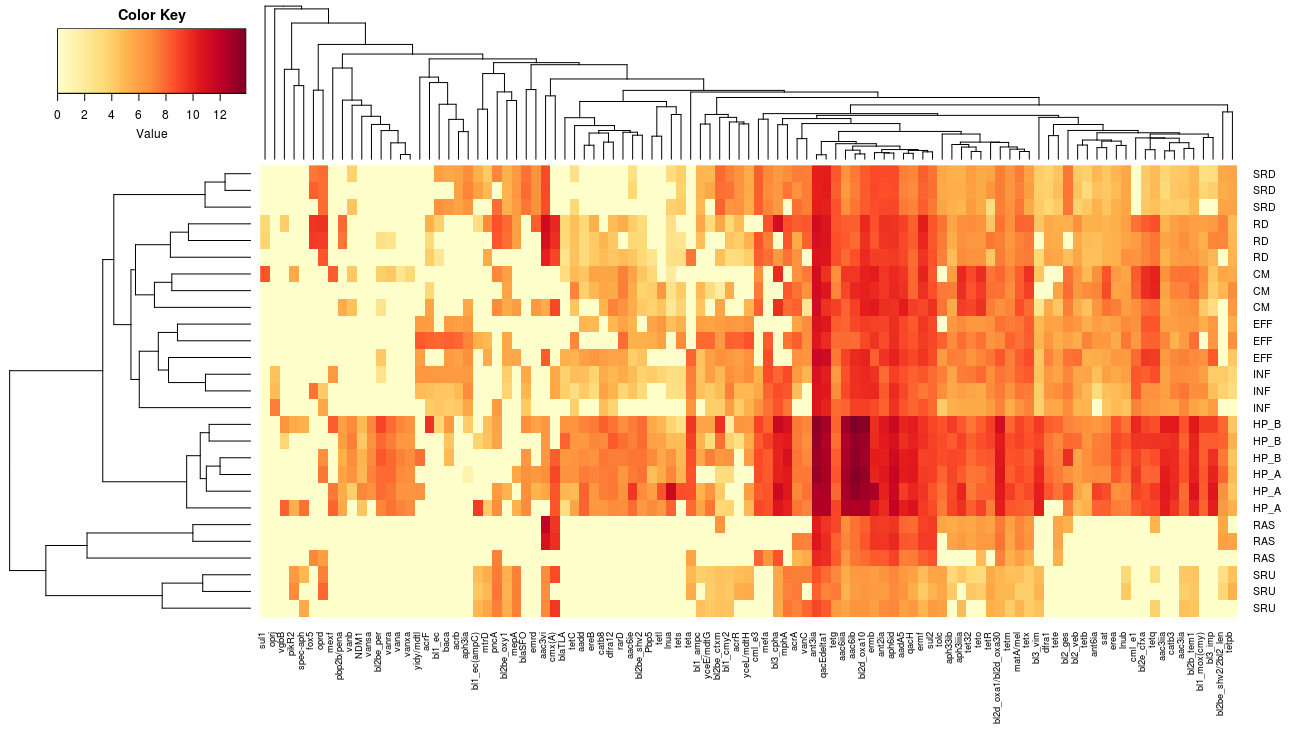
\

**Figure S-13:** Procrustes test depicting significant correlations between ARGs content and bacterial composition (16S rRNA gene OTUs data) on the basis of Bray−Curtis dissimilarity metrics *(sum of squares M12 = 0.3445 , r = 0.8096, P = 0.001, 999 permutations). Sample codes: SRU) sediment river upstream, CM) community-associated wastewater, HP) hospital associated wastewater, INF) WWTP Influent, RAS) recycled activated sludge, EFF) WWTP effluent, RD) water column river downstream, SRD) Sediment river downstream.*


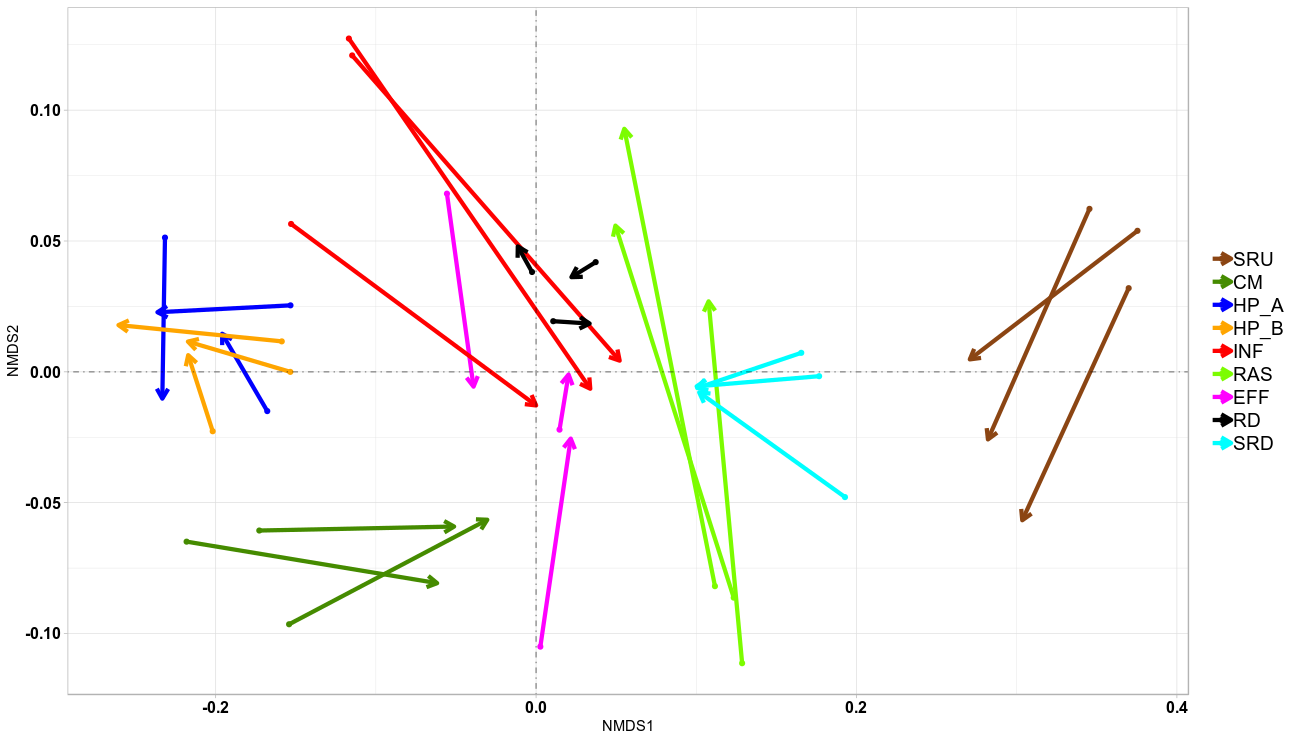


**Figure S-14:** The network analysis revealing co-occurrence patterns among ARG subtypes, taxa (order level), transposons and integrons. A connection represents a strong (Spearman’s correlation coefficient p > 0.8) and significant (P-value > 0.01) correlation. The size of each node is proportional to the number of connections; i.e., the degree. Correlation networks show two groups: A) composed of ARGs, transposases, integrases and taxa related with WWTP influents (see Figure 6), and B) only contain taxa associated with the RAS.


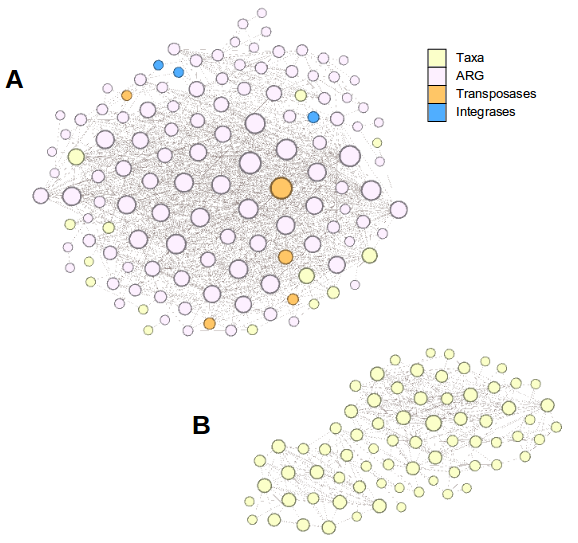


**Figure S-15:** Group B of the network analysis from Figure S-11. This group contain taxa associates with the RAS such as Acidimicrobiales, Actinomycetales, Alteromonadales, Bacillales, Caulobacteriales, Cytophagales, Gaiellales, Legionellales, Myxococcales, PHOS-HD29, Rhizobiales, Rhodobacteriales, Rhodospirillales, Saprospirales, SC-I-84, Solirubrobacterales, Sphingobacteriales, Spingomonadales and Xanthomonadales.
